# Overall survival and toxicities regarding thoracic three-dimensional radiotherapy with concurrent chemotherapy for stage IV non-small cell lung cancer: results of a prospective single-center study

**DOI:** 10.1186/1471-2407-13-474

**Published:** 2013-10-12

**Authors:** Sheng-Fa Su, Yin-Xiang Hu, Wei-Wei Ouyang, Bing Lu, Zhu Ma, Qing-Song Li, Hui-Qin Li, Yi-Chao Geng

**Affiliations:** 1 Department of Thoracic Oncology, Affiliated Hospital of Guiyang Medical College, and Guizhou Cancer Hospital, 1 Beijing Road West, Guizhou, Guiyang, People’s Republic of China

**Keywords:** Non-small cell lung cancer, Stage IV, Concurrent chemoradiotherapy, Thoracic three-dimensional radiotherapy, Overall survival

## Abstract

**Background:**

The role of chemotherapy given concurrently with thoracic three-dimensional radiotherapy for stage IV non-small cell lung cancer (NSCLC) is not well defined. We performed this study to investigate overall survival and toxicity in patients with stage IV NSCLC treated with this modality.

**Methods:**

From 2003 to 2010, 201 patients were enrolled in this study. All patients received chemotherapy with concurrent thoracic three-dimensional radiotherapy. The study endpoints were the assessment of overall survival (OS) and acute toxicity.

**Results:**

For all patients, the median survival time (MST) was 10.0 months, and the 1-, 2- and 3-year OS rates were 40.2%, 16.4%, and 9.6%, respectively. The MST was 14.0 months for patients who received a total radiation dose ≥63 Gy to the primary tumor, whereas it was 8.0 months for patients who received a total dose <63 Gy (*P* = 0.000). On multivariate analysis, a total dose ≥63 Gy, a single site of metastatic disease, and undergoing ≥4 cycles of chemotherapy were independent prognostic factors for better OS (*P* = 0.007, *P* = 0.014, and *P* = 0.038, respectively); radiotherapy involving metastatic sites was a marginally significant prognostic factor (*P* = 0.063). When the whole group was subdivided into patients with metastasis at a single site and multiple sites, a higher radiation dose to the primary tumor remained a significant prognostic factor for improved OS. For patients who received ≥4 cycles of chemotherapy, high radiation dose remained of benefit for OS (*P* = 0.001). Moreover, for the subgroup that received <4 chemotherapy cycles, the radiation dose was of marginal statistical significance regarding OS (*P* = 0.063). Treatment-related toxicity was found to be acceptable.

**Conclusions:**

Radiation dose to primary tumor, the number of metastatic sites, and the number of chemotherapy cycles were independent prognostic factors for OS in stage IV NSCLC patients treated with concurrent chemoradiotherapy. In addition to systemic chemotherapy, aggressive thoracic radiotherapy was shown to play an important role in improving OS.

**Trial registration:**

Registered on (ChiCTR-TNC-10001026)

## Background

For non-small cell lung cancer (NSCLC) patients with stage IV disease and a good performance status, platinum-based combination therapy improves survival and quality of life [1]. However, survival time has not been obviously increased using chemotherapy over the past 10–15 years [2]. Systemic chemotherapy is the standard treatment modality for stage IV NSCLC, and thoracic radiotherapy is most typically used for palliation [1,3-5]. Thoracic radiotherapy is an effective way to relieve symptoms (hemoptysis, cough, chest pain, dyspnea, and others) that are caused by the locoregional growth of tumor, and can improve the survival of patients with better performance status using higher radiation doses [4,5]. Wagner et al. [6] pointed out that one of the limitations of published studies concerning patients receiving radiation therapy for stage IV NSCLC is the lack of data on the use of chemotherapy in these patients.

There is increasing evidence that selected patients with stage IV disease could benefit from aggressive thoracic radiotherapy beyond palliative irradiation [7-9]. However, most of the published data include only small patient numbers, and two-dimensional radiation therapy (2D-RT) has commonly been used. Moreover, the role of chemotherapy given concurrently with thoracic radiotherapy for stage IV NSCLC patients is not well defined; concurrent chemoradiotherapy is not recommended as routine treatment modality, with the exception of clinical trials [5]. Given these concerns, we sought to determine if concurrent chemotherapy and thoracic three-dimensional conformal radiotherapy (3D-CRT) or intensity-modulated radiotherapy (IMRT) are safe, efficient and feasible treatment modalities for patients with stage IV NSCLC. Thus, we performed this prospective study to investigate patient survival and toxicity regarding this treatment modality in our single institution (Additional file 1).

## Methods

### Patient selection and pretreatment evaluation

Since January 2003, patients with stage IV NSCLC that fulfilled all of the following criteria have been treated using a prospective institutional protocol at the Affiliated Hospital of GuiYang Medical College, and Guizhou Cancer Hospital China. The inclusion criteria were as follows: 1) histologically or cytology confirmed NSCLC; 2) newly diagnosed stage IV disease according to the staging system of the 2002 American Joint Committee on Cancer (AJCC); 3) an age of 18–80 years; 4) a Karnofsky Performance Status score ≥70%; 5) adequate bone marrow, liver and renal function; neutrophils ≥ 1.5 × 10^9^/L, platelets 80 × 10^9^/L, hemoglobin ≥80 g/L, AST and ALT ≤2× the upper limit of the institutional normal range, total bilirubin ≤1.25× the upper limit of the institutional normal range, and creatinine concentration ≤120 μmol/L; 6) no contraindication for radiotherapy and chemotherapy; 7) limited metastatic disease (≤5 sites); 8) patients were expected to receive thoracic radiotherapy at a dose of ≥40 Gy in 20 fractions. The exclusion criteria were as follows: 1) a history involving thoracic surgery, radiotherapy or chemotherapy; 2) pregnancy or lactation; 3) previous malignancy or other concomitant malignant disease. This prospective study was reviewed by the ethical review boards in China (Ethics Committee of the Affiliated Hospital of Guiyang Medical University, GuiYang, China), and the informed consent for treatment was obtained from all patients.

Pretreatment evaluation included a complete physical examination and hematologic and biochemistry profiles. Examinations using fiberoptic bronchoscopy and contrast-enhanced computed tomography (CT) of chest were performed to accurately evaluate the extent of the primary tumor and regional lymph nodes. Bone scintigraphy, contrast-enhanced CT of the abdominal region, and magnetic resonance imaging of the head were routinely used to detect distant metastases. Additional investigations were performed if indicated.

### Thoracic radiotherapy protocol

All patients were immobilized in the supine position using a T bar, wing board, and Vac-lock cradle. Images with contrast were obtained from the CT simulator for treatment planning purposes. All patients were scanned using serial 5-mm slices from the hyoid bone through to the third lumbar vertebra. All patient 3D-CRT or IMRT treatment plans were performed using the ADAC pinnacle planning system (version 7.4f) and dose distribution was computed with a tissue heterogeneity correction. The gross tumor volume (GTV) included thoracic primary tumors and the visible mediastinal lymph nodes on the treatment planning CT scan; the planning target volume (PTV) was defined as the GTV plus a 1.5-cm margin for setup uncertainty and respiratory motion. Radiation was delivered using a linear accelerator that generated 6 MV photons. The V20 (percentage of the total lung volume receiving ≥20 Gy), the maximal point dose for the spinal cord and the mean esophagus dose were required to be ≤32%, ≤50 Gy and ≤35 Gy, respectively, for individual treatment plans.

Patients received late-course accelerated hyperfractionated radiotherapy (LCAHRT) to the thoracic primary site using 3D-CRT or IMRT techniques. The first course of radiotherapy was given in 2 Gy fractions, 5 days a week to a total dose of 40 Gy; LCAHRT was delivered in two fractions of 1.5 Gy each with an interval of 6–8 h per day. It was decided to deliver a prescription dose of 60–70 Gy to patients; if individual tolerability was not acceptable, lower doses of ≥40 Gy would be given. The PTV should be covered by at least the 90% isodose surface. At acceptable radiation doses to the normal tissue, radiation dose to the primary thoracic tumor can be escalated to 74 Gy. Thoracic radiation treatment was implemented concurrently with chemotherapy.

### Chemotherapy protocol

Platinum-based doublets chemotherapy was used for all patients; the selection of regimens was in accordance with prior studies [10,11]. Concurrent thoracic radiation was given within 1 week following the start of chemotherapy. The commonly used regimens and usage were as follows: 140 mg/m^2^ of paclitaxel (P) or 75 mg/m^2^ of docetaxel (D) on day 1, followed by 80 mg of cisplatinum (C) per square meter of body-surface area (mg/m^2^) or carboplatin (Cb) at a dose calculated to produce an area under the concentration-time curve of 6.0 mg/ml/min were administrated on day 2; and vinorelbine (V) was administered at a dose of 25 mg/m^2^ on days 1 and 8 during thoracic radiotherapy every 21 days. After completion of thoracic radiotherapy, patients demonstrating a response or stable disease continued chemotherapy up to 4–6 cycles, whereas patients who experienced progressive disease or unacceptable toxicity were transferred to second-line therapy. Platinum and taxane-based chemotherapy were the main regimens used in the current study; the number of patients who received P and C or P and Cb, D and C or D and Cb, and V and C arms was 83, 103, and 15, respectively. The total number of cycles delivered for all patients was 617 (mean number per patient, 3.0).

### Evaluation of treatment-related toxicity and response

Treatment-related acute toxicity was scored according to the National Cancer Institute’s Common Terminology Criteria for Adverse Events (CTC) version 3.0. During the course of treatment, a routine blood test was performed at least once per week; and routine blood, liver function, and renal function tests and electrocardiograms were examined prior to chemotherapy. If necessary, chest X-ray or CT examination and barium meal radiography were used to evaluate radiation pneumonitis and esophagitis. Treatment response was assessed by extramural reviewers using the Response Evaluation Criteria in Solid Tumors (RECIST).

### Follow-up and statistical analysis

After the completion of treatment, the patients were subsequently followed up monthly for the first 3 months, every 3 months for 2 years, and then every 6 months. Intent-to-treat analyses were performed on data from all patients who entered the study. The endpoints of this study included overall survival (OS) and acute toxicity. The overall survival time was measured from the first day of concurrent chemoradiotherapy to the date of death or the last follow-up. The Statistical Package for Social Sciences, version 13.0 (SPSS, Chicago, IL, USA) was used for statistical analysis. The Kaplan-Meier method was used to calculate the OS. The log-rank test was used to compare the survival curves. Multivariate Cox regression analysis was used to test independent significant prognostic factors for OS. All statistical tests were two-sided, and a *P* value <0.05 was considered as being statistically significant.

## Results

### Survival analysis

From January 2003 to July 2010, a total of 201 cases were enrolled in this study. The clinical characteristics are listed in Table 1. The most common site of metastatic disease at diagnosis was the bone (52% of patients); 69 (34%) patients had lung metastasis and 55 (27%) had metastasis in brain. One hundred and twenty-one (60%) patients had metastasis in only one site, 53 in the bone, 25 in the lung, 22 in the brain, 5 in the liver, 5 in the adrenal glands, and 11 in other locations. The median follow-up period was 9.5 (range, 1–55) months. A median radiation dose of 63 (range, 30–72) Gy was delivered to the primary tumor. For the whole group, 18 patients received <40 Gy to the primary tumor; among these 18 patients, 10 gave up treatment following radiotherapy on personal grounds, and eight refused following radiotherapy because of emerging new metastasis. In total, 98 patients received radiotherapy concurrently or sequentially with chemotherapy for metastatic lesions in 3–10 Gy daily fractions to a total dose of 20–60 Gy.

**Table 1:** Clinical characteristics (201 patients)

**Characteristic**		**Median (range)**	**No.**	**%**
Gender				
Male			150	74.6
Female			51	25.4
Age (years)		60 (28–80)		
<60			99	49.3
≥60			102	50.7
Pathological type				
Squamous carcinoma			67	33.3
Adenocarcinoma			108	53.7
others			26	13.0
T stage				
T_1-2_			74	36.8
T_3-4_			127	63.2
N stage				
N_0-1_			35	17.4
N_2-3_			166	82.6
Prescribed dose		63 (22–72)		
<63Gy			108	53.7
≥63Gy			93	46.3
No. of chemotherapy cycle		4 (1–5)		
1			19	9.5
2			45	22.4
3			44	21.9
4			89	44.3
5			4	2.0
No. of metastatic sites		1 (1–5)		
1			121	60.2
2			55	27.4
3			20	10.0
4			4	2.0
5			1	0.5
Response of primary tumor				
Complete response			12	6.0
Partial response			113	56.2
Stable			40	19.9
Progressive			16	8.0
Unassessable			20	10.0
Radiotherapy to metastasis				
Patients with 1 metastatic site	Yes		76	62.8
	No		45	37.2
Patients with ≥2 metastatic sites	Yes		22	27.5
	No		58	72.5

**Figure 1:**
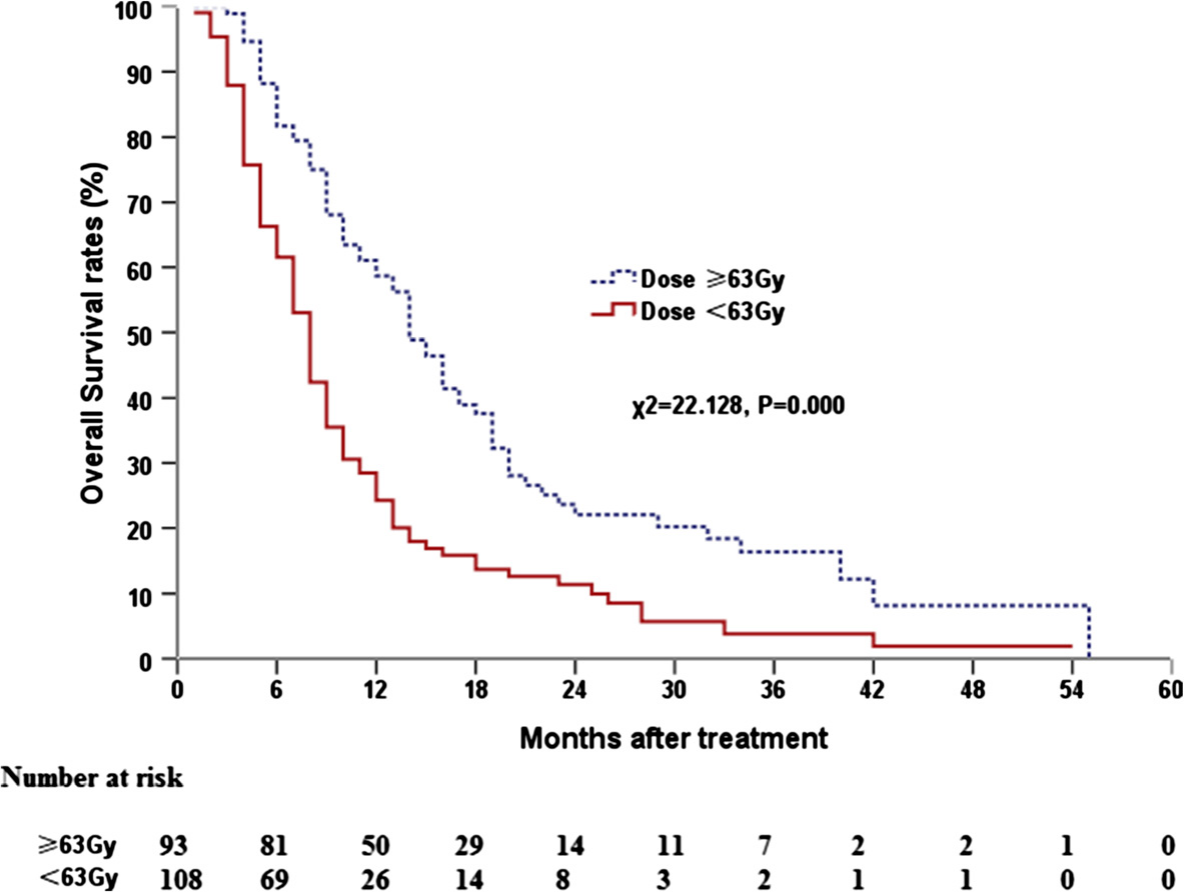
Comparison of dose–response curves for overall survival at different radiation doses.

For all patients, the median survival time (MST) was 10.0 months (95% CI, 8.50–11.50), and the 1-, 2-, and 3-year OS rates were 40.2%, 16.4%, and 9.6%, respectively. The MST, and 1-, 2-, and 3-year OS rates were 14.0 (95% CI, 11.86–16.14) months, and 58.7%, 22.0%, and 16.3%, respectively for patients who received a thoracic radiation dose ≥63 Gy; whereas the MST was 8.0 (95% CI, 7.01–8.99) months, and 24.2%, 11.4%, and 3.8%, respectively for patients who received a dose <63 Gy. The difference between the groups that received ≥63 Gy and <63 Gy was statistically significant (*P* = 0.00; Figure 1). Patients who received radiotherapy for metastasis had a longer MST than those who did not receive radiotherapy for metastasis (13.0 months vs. 8.0 months; *P* = 0.003).

Contrast-enhanced CT of the chest was performed to evaluate the treatment response of the primary tumor. Chest X-ray was not used to evaluate the treatment response and local control of the primary tumor. In total, 90% (181/201) of patients received CT examination at 1 month after completion of thoracic irradiation, and 74.6% (150/201) patients received further CT examination of the chest after 1 month. At 1 month after thoracic radiotherapy, 69% (125/181) of patients were confirmed to have responded to the treatment, including 6.6% (12/181) of patients with a complete response (CR), 62.4% (113/181) with a partial response (PR), 22.1% (40/181) with stable disease (SD), and 8.8% (16/181) with progressive disease (PD). Among these 181 patients, there was a significant association between the treatment response of the primary tumor and survival; patients with a response (CR + PR) had a longer MST than those without a response (SD + PD) (13.0 months vs. 7.0 months; *P* = 0.001). Patients receiving ≥63 Gy to the primary tumor had higher rates of treatment response as compared with those receiving <63 Gy (84.9% vs. 52.3%; *P* = 0.000). With regard to multivariate analysis, patients with a treatment response (HR, 0.88; *P* = 0.005) had an improved OS.

**Figure 2:**
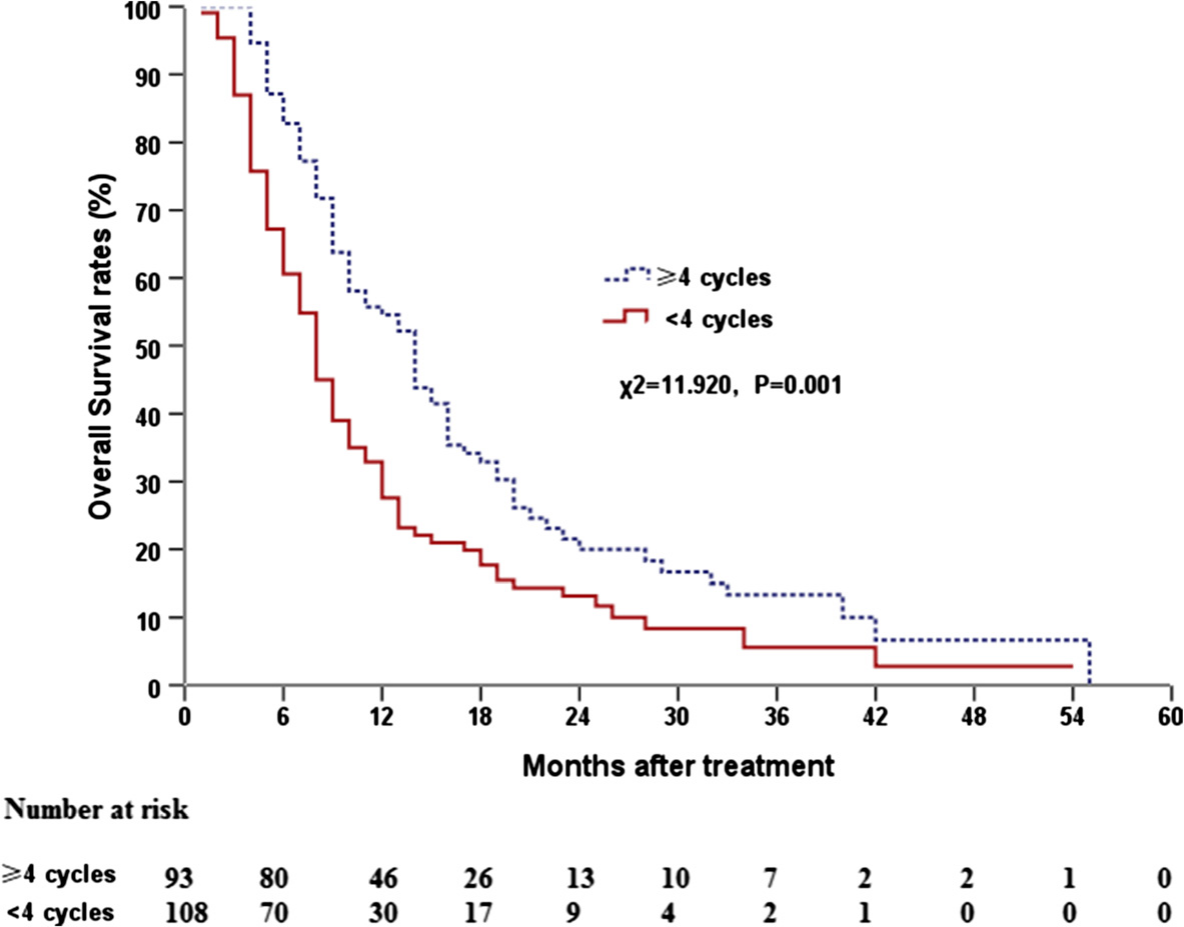
Comparison of overall survival curves with regard to different chemotherapy cycles.

**Figure 3:**
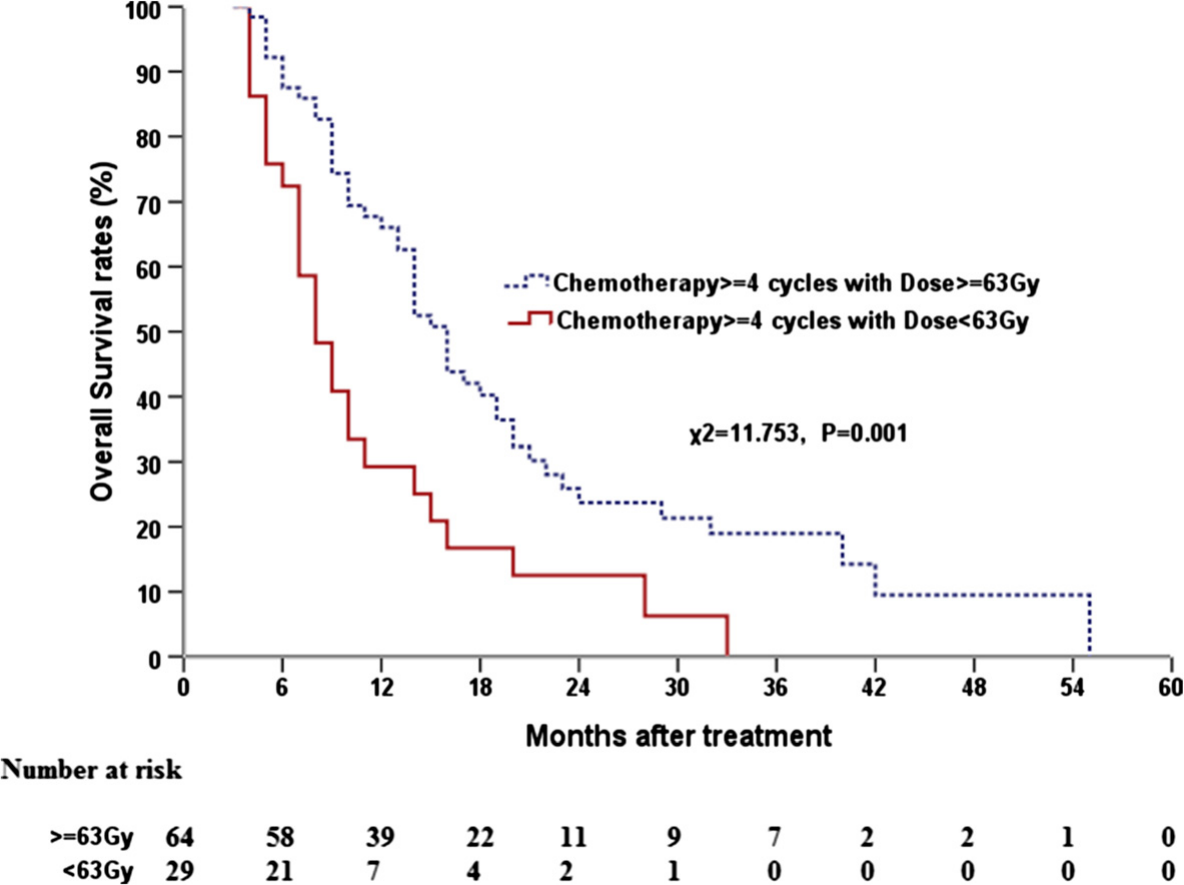
**Comparison of dose–response curves for overall survival at different radiation doses for patients treated with** ≥**4 chemotherapy cycles.**

OS was significantly prolonged in patients who received ≥4 cycles of chemotherapy relative to those who received <4 cycles; the MSTs for these two groups were 14.0 months (95% CI, 11.37–16.63) and 8.0 months (95% CI, 6.67–9.33; *P* = 0.001; Figure 2), respectively. For the subset of patients who received ≥4 cycles, the survival time differed significantly between patients that had received a radiation dose ≥63 Gy and those that had received a radiation dose <63 Gy (*P* = 0.001; Figure 3); the MSTs for these two groups were 16 months (95% CI, 14.00–18.00) and 8 months (95% CI, 5.95–10.05), respectively. For patients who received <4 cycles, this difference between patients that had received a radiation dose ≥63 Gy and those that had received a radiation dose <63 Gy was of marginal statistical significance (*P* = 0.063).

**Figure 4:**
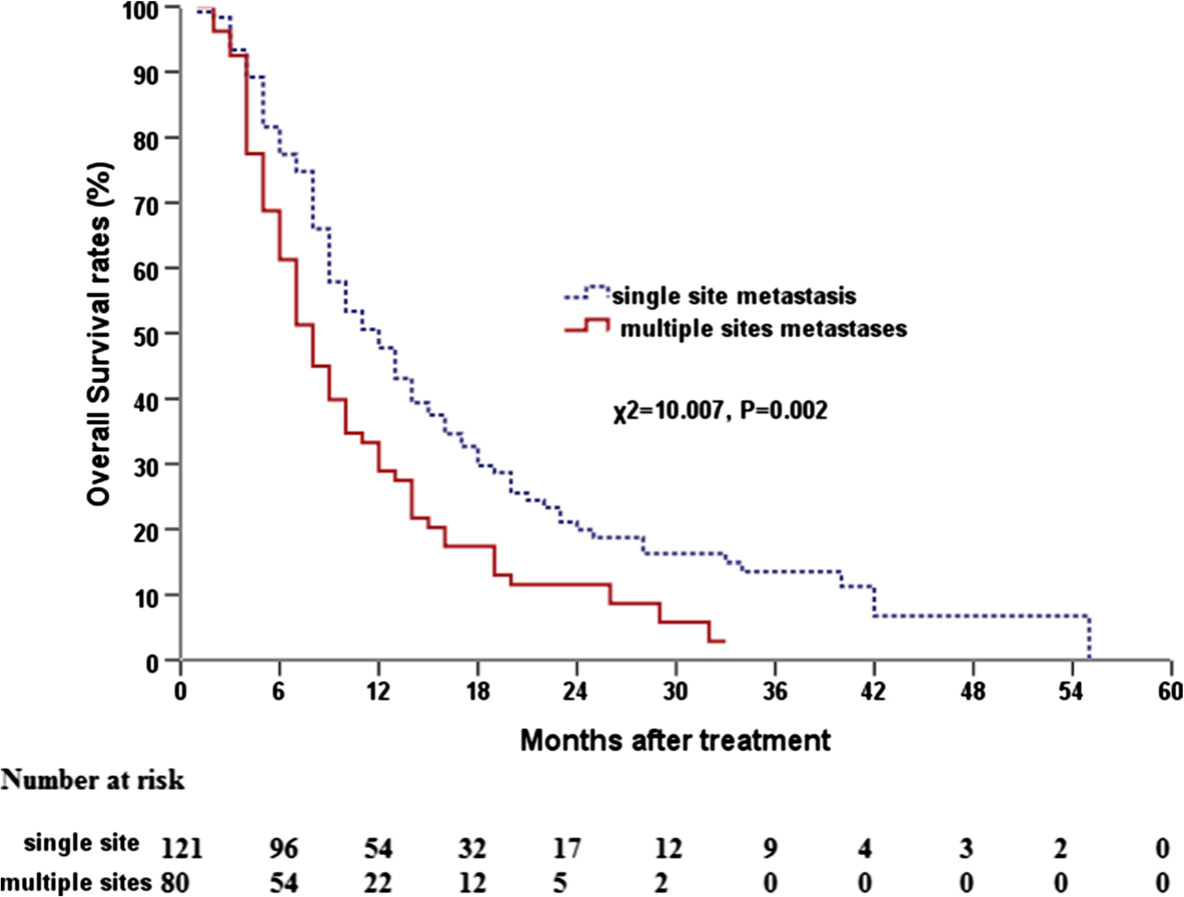
Comparison of overall survival curves between single site metastases and multi-sites metastases.

The MST for patients with a single metastatic site was 12.0 months (95% CI, 9 · 50–14 · 50) whereas for patients with ≥ 2 metastatic sites it was 8.0 months (95% CI, 6.85–9.34; *P* = 0.002; Figure 4). For the subset with single site metastasis, the MST for patients that had received a radiation dose ≥63 Gy to the primary tumor was 17.0 months (95% CI, 13.0–21.0) whereas it was 9.0 months (95% CI, 7.0–11.0) for patients that had received a dose <63 Gy (*P =* 0.001). The location of metastasis (brain, bone, or other locations) was not related to OS (*P* = 0.213). When the whole group was subdivided into patients with metastasis at a single site and multiple sites, multivariate analysis showed that patients receiving a radiation dose ≥63 Gy to the primary tumor had a significantly better OS (Table 2).

**Table 2:** Multivariate analysis of parameters for the prediction of overall survival

**Variable**	**All patients**		**Patients with single sites of metastasis**		**Patients with** ≥**2 sites of metastasis**	
	**HR**	***P *value**	**HR**	***P *value**	**HR**	***P *value**
Sex(female vs. male)	0.742	0.117	0.605	0.079	0.786	0.201
Age(<60y vs. ≥60y)	0.841	0.288	0.915	0.740	0.856	0.340
Pathological type (squamous vs. non-squamous )	0.991	0.957	0.919	0.767	0.973	0.872
T stage(T3-4 vs. T1-2)	1.109	0.537	1.178	0.667	1.188	0.296
N stage(N2-3 vs. N0-1)	1.286	0.232	1.213	0.597	1.305	0.208
Thoracic radiation dose	0.615	0.007	0.576	0.045	0.619	0.008
(≥63Gy vs. <63Gy)						
Radiotherapy to metastases(yes vs. no)	0.729	0.063	0.340	0.001	0.674	0.017
Chemotherapy cycles	0.693	0.038	0.625	0.079	0.691	0.039
(≥4 cycles vs. < 4 cycles)						
Metastasis status	1.513	0.014	-	-	-	-
(multi- vs. single)						

Univariate analysis showed that pathological type, gender, age, T-stage, and N-stage were not associated with OS. For the whole group, multivariate analysis indicated that a radiation dose ≥63 Gy to the primary tumor, a single site of metastatic disease, and a number of chemotherapy cycles ≥4 were independent prognostic factors for better OS; radiation to metastatic sites was marginally significant in this regard (Table 2).

### Treatment toxicity

For the whole group, the incidence of acute Grade 2 to Grade 3 gastrointestinal toxicity was 45.2%, and no Grade 4 or 5 gastrointestinal toxicity was observed. Hematologic toxicity was the most common and severe complication. The incidence of acute Grade 3 to 4 leukocytes, thrombocytopenia, and anemia was 34.8%, 17.9%, and 15.4%, respectively. The most frequently observed acute pneumonitis or esophagitis was mainly Grade 0 or Grade 1. The incidence of acute Grade 2–3 pneumonitis and esophagitis was 9.5% and 13.5%, respectively. Table 3 shows the toxicity data for the whole group.

**Table 3:** Incidence of acute toxicity (201 patients)

**Adverse effects**	**Grade 0 to 1**	**Grade 2**	**Grade 3**	**Grade 4**
Gastrointestinal	110 (54.7)	64(31.8)	27(13.4)	0 (0.0)
Leukocytes	67 (33.3)	64(31.8)	48(23.9)	22(10.9)
Platelet	145 (72.1)	20(9.9)	26(12.9)	10(5.0)
Hemoglobin	120(59.7)	50(24.9)	21(10.4)	10(5.0)
Pneumonitis	182(90.5)	11(5.5)	8(4.0)	0(0.0)
Esophagitis	174(86.6)	24(12.0)	3(1.5)	0(0.0)

## Discussion

In the present series, we sought to determine whether or not thoracic 3D-CRT using concurrent third-generation chemotherapy regimens could improve OS for patients with stage IV NSCLC. As compared with historical data concerning patients who had been treated with third-generation chemotherapy regimens or newer agents (e.g. pemetrexed) [10-12], the survival times of patients in the current study were not decreased. We found that the system of chemotherapy involved in the thoracic 3D-CRT used in our study was a safe and feasible treatment modality for patients with stage IV NSCLC. Our results also suggested that radiation doses ≥63 Gy to the primary tumor, a single site of metastatic disease, radiotherapy for metastatic sites and >4 cycles of chemotherapy were independent prognostic factors for better OS in stage IV NSCLC patients treated with concurrent chemoradiotherapy.

Fairchild et al. [4] reported the results of a systematic review of 13 randomized controlled trials involving palliative thoracic radiation, and found that an improvement in survival and symptoms was seen with higher-dose radiation schedules as compared with lower dose radiation schedules. Lopez et al. [7] suggested that the delivery of at least 63 Gy to the primary tumor was associated with improved OS in patients with oligometastatic NSCLC at diagnosis. The results of our analysis of thoracic radiotherapy concur with the findings of Fairchild et al. [4] and Lopez et al. [7], namely that higher-dose radiation treatment had a greater likelihood of improving survival.

There was a limitation to the current study, in that consistent imaging data were not gained in a proportion of patients for the evaluation of the relationship between OS and local-regional control (LCR). Several publications have confirmed that higher radiation doses are associated with improved local tumor control and OS in patients with NSCLC [7,13]. Although we did not obtain data regarding LCR in our study, the results indicate that higher radiation dose to the primary tumor and treatment response were prognostic factors for better OS. From the results of the present study, we speculate that aggressive local therapy to primary tumor can improve OS. A retrospective analysis by Lopez et al. [7] also indicated that aggressive local therapy to the primary tumor can improve OS for patients with oligometastatic NSCLC.

Consistent with the conclusion of a study by Scagliotti et al. [10], we found that the number of distant metastatic sites was associated with OS. However, it is worthwhile noting that higher radiation dose delivery to the primary tumor and radiotherapy for metastatic sites were independent prognostic factors for better OS, when patients with metastasis in single site and those with multiple sites were analyzed separately. Our results indicated that aggressive radiation treatment at the primary tumor and metastatic site translated to improved OS, whether or not patients had metastasis at a single site or multiple sites. Several recent publications have also shown that patients with limited metastases may benefit from radiotherapy for the primary tumor and distant metastasis [7-9].

A consensus on the use of chemotherapy administered concurrently with radiotherapy for stage IV NSCLC has not been reached. The findings from a retrospective study by Lopez et a1. [7] revealed that higher radiation doses to the primary tumor are associated with improved OS in patients with oligometastatic NSCLC; most of the patients (67%) received platinum and taxane-based concurrent chemotherapy. These authors found [7] that patients that received concurrent chemoradiotherapy had a trend involving improved OS relative to those that did not undergo chemoradiotherapy (P = 0.055). We searched the PubMed data base thoroughly and found only one randomized phase III study that had directly assessed whether or not patients with advanced NSCLC would benefit from chemotherapy administered concurrently with thoracic palliative radiotherapy [14]. The randomized phase III study by Ball et al. [14] revealed that the addition of chemotherapy to radiotherapy resulted in no improvement in survival; the MST was 6.8 months in patients treated with chemoradiotherapy, whereas it was 6.0 months in patients who were treated with radiation alone. It is worthwhile to notinge that fluorouracil, which is an outmoded agent that is rarely used in systemic therapy for NSCLC, was administered concurrently with radiotherapy as a palliative treatment for intrathoracic disease using 2D-radiotherapy in a study by Ball et al. [14]. Our results showed that a higher radiation dose to the primary tumor concurrent with chemotherapy for stage IV NSCLC resulted in a considerable improvement in survival. As compared with the study by Ball et al. [14]*,* patient survival in our study was improved*.* This may have been due in part to the following reasons. Firstly, newer agents such as P, D, and V were used in our study; these agents have been identified as improving survival [15]. Secondly, the use of modern radiotherapy technologies (IMRT or 3D-CRT) with higher doses (median dose, 63 Gy) have been used to improve the local control of thoracic tumors; this has contributed to a reduction in the death rate caused by locoregional growth of tumor and decreased the sources of metastasis.

Although we used different chemotherapy regimens for patients, they had similar response rates and survival times in the treatment of advanced NSCLC [10,16]. In the current study, the effect of the number of chemotherapy cycles on OS was also found to be statistically significant using multivariate analysis. The recommended number of chemotherapy cycles for stage IV NSCLC was 4–6 according to the ASCO guidelines [3]. We found that when patients were grouped according to the number of chemotherapy cycles, those that received ≥4 cycles of chemotherapy exhibited a prolongation of survival time. Moreover, for the subgroup that received ≥4 cycles of chemotherapy, we observed that higher radiation doses (≥63 Gy) to the primary tumor could improve OS. For the subgroup that received <4 cycles of chemotherapy, there was a trend for improved OS at higher radiation doses. In patients with stage IV NSCLC, thoracic irradiation is most typically delivered for palliation [3-5]. Our findings indicated that patients can benefit from aggressive radiation treatment (≥63 Gy) to the primary tumor based on the delivery of a sufficient number of cycles (≥4 cycles) of systemic chemotherapy. There was no randomization in the current trial. Further evidence is needed to determine whether or not thoracic radiotherapy with concurrent chemotherapy is superior to chemotherapy alone, and if NSCLC patients with limited metastasis can benefit from higher radiation doses.

The use of concurrent chemoradiotherapy is not recommended for advanced NSCLC. This is because of one important factor, namely that toxicity is increased by concurrent chemoradiotherapy relative to radiation alone. Concurrent chemoradiotherapy has become a standard of treatment for patients with unresectable locally advanced NSCLC. The incidence of toxicity in the current study (including hematologic and non-hematologic toxicity) was not increased, as compared with studies of concurrent treatment with radiotherapy and chemotherapy in locally advanced NSCLC [17]. As far as toxicity is concerned, concurrent chemoradiotherapy could be acceptable for stage IV NSCLC.

## Conclusions

Patients with stage IV NSCLC with good performance status who were treated with higher radiation doses (≥63 Gy) to the primary tumor concurrently with systemic chemotherapy had improved survival outcomes with acceptable toxicity; this was especially true for patients with single metastatic sites. Patients can benefit from higher radiation doses to the primary tumor, whether they have metastasis at a single site or multiple sites. We found that among patients who had undergone ≥4 cycles of chemotherapy, those that had received higher radiation doses (≥63 Gy) to the primary tumor benefited. For the subgroup who received <4 cycles of chemotherapy, higher radiation doses had a trend that involved improved OS. Our findings suggest that patients benefit from higher radiation doses (≥63 Gy) to the primary tumor based on their having received a sufficient number of cycles (≥4) of systemic chemotherapy. In addition to systemic chemotherapy, clinicians should consider aggressive thoracic radiotherapy beyond palliative intent.

## Competing interests

The authors declared that they have no competing interests.

## Authors’ contributions

BL designed the study. S-FS, Y-XH, W-WO, ZM, Q-SLi, H-QL, and Y-CG collected the data. BL, S-FS, Y-XH, and W-WO undertook the data analysis and interpretation, and wrote the report. BL and S-FS carried out the statistical analysis. All authors read and approved the final manuscript.

## Pre-publication history

The pre-publication history for this paper can be accessed here:

http://www.biomedcentral.com/1471-2407/13/474/prepub

## Supplementary Material

Additional file 1 Registration of the study. Click here for file
