# Supplementary material for: Overall survival and toxicities regarding thoracic three-dimensional radiotherapy with concurrent chemotherapy for stage IV non-small cell lung cancer: results of a prospective single-center study
Source: BMC Cancer. 2013 Oct 12;13:474. doi: 10.1186/1471-2407-13-474 (PMC3852781; doi:10.1186/1471-2407-13-474)
Supplement: Additional file 1 — Registration of the study. [file 1471-2407-13-474-S1.docx]

Dear editor,

The following shows registration process of our clinical trial.

We performed current prospective study since January 2003, and reported preliminary outcomes of 119 patients at annual meeting of Chinese society for radiation oncology in September 2009[1]. However, we did not registered trial number for this prospective study before September 2009. We sponsored multicenter to join this clinical study and registered on www.chictr.org (trial registration number: ChiCTR-TNC-10001026) in 2009. From January 2003 to July 2010, totally, 201 patients were included in this prospective study in our single-centre. We reported the outcomes of these 201 patients in this manuscript.

1. 卢冰, 付和谊, 欧阳伟炜, 苏胜发, 胡银祥, 甘家应, 吴伟莉, 栗蕙芹: Ⅳ期NSCLC化疗同期胸部三维放射治疗的临床研究. 中华医学会放射肿瘤治疗分会六届二次暨中国抗癌协会肿瘤放疗专业委员会二届二次学术会议论文集. 2009.[Chinese]

1. Bing Lu, HeYi Fu, WeiWei OuYang, ShengFa Su, YingXiang Hu, JiaYing Gan, WeiLi Wu, HuiQing Li: Clinical study of thoracic three dimensional radiotherapy with concurrent chemotherapy for stage Ⅳ non-small cell lung cancer. Affiliated Chinese society for radiation oncology of Chinese medical association. 2009.

Sincerely Yours,

Bing Lu, MD.

Department of Thoracic Oncology, Cancer Hospital of GuiZhou Province, 1 Beijing Road West, Guiyang, Guizhou, People's Republic of China. Tel: +86-851-6513076,

E-mail: lbgymaaaa@sohu.com
